# PhIP-Seq: unveiling the complexity of antibody repertoires in health and disease

**DOI:** 10.3389/fimmu.2026.1735735

**Published:** 2026-02-10

**Authors:** Wenjie Tang, Qijing Gai, Junjie Yang, Jianqing Chen, Zhengbing Lyu

**Affiliations:** 1 Department of Biopharmacy, College of Life Sciences and Medicine, Zhejiang Sci-Tech University, Hangzhou, China; 2 Zhejiang Q-peptide Biotechnology Co., Ltd, Shaoxing, China

**Keywords:** autoimmune diseases, cancer immunology, infectious diseases, phage display, PhIP-Seq

## Abstract

Phage-Immunoprecipitation Sequencing (PhIP-Seq) merges phage display with next-generation sequencing to enable high-throughput profiling of antibody repertoires. This review synthesizes the technical evolution of the PhIP-Seq platform, critically assessing the workflow from peptide library design and immunoprecipitation to bioinformatics analysis. We evaluate strategies for optimizing library diversity and minimizing non-specific binding, while addressing inherent limitations such as the detection of conformational epitopes and post-translational modifications. The clinical utility of PhIP-Seq is examined through its application in identifying novel autoantigens in systemic lupus erythematosus and multiple sclerosis, mapping viral epitopes in SARS-CoV-2 and Plasmodium falciparum, and detecting tumor-associated antigens. Finally, we discuss the trajectory of the field toward integration with multi-omics datasets and the development of point-of-care diagnostic tools.

## 1 Introduction

Since its introduction by Larman and colleagues in 2011, Phage-Immunoprecipitation Sequencing (PhIP-Seq) has provided a scalable method for dissecting antibody-antigen interactions (1). Unlike conventional serological assays such as ELISA and Western blotting, which are often limited by throughput and a narrow antigen scope (2, 3), PhIP-Seq facilitates the concurrent screening of peptide arrays numbering in the millions (4, 5). By interrogating synthetic representations of proteomes, the platform provides a granular view of humoral immunity.

The technology has been applied to delineate disease biomarkers across diverse pathologies. In systemic lupus erythematosus (SLE), PhIP-Seq has linked specific IgG autoantibodies to myocardial dysfunction (6, 7). In infectious diseases, it has been utilized to map antibody landscapes against SARS-CoV-2 and identify correlates of post-acute sequelae (8, 9). Furthermore, the platform supports large-scale seroepidemiological surveys to assess population-level pathogen exposure (9–11). This review critically examines the methodological foundations of PhIP-Seq, synthesizes current technical challenges, and evaluates its expanding clinical applications.

## 2 The PhIP-Seq technical framework: methodology, optimization, and challenges

**Figure 1:**
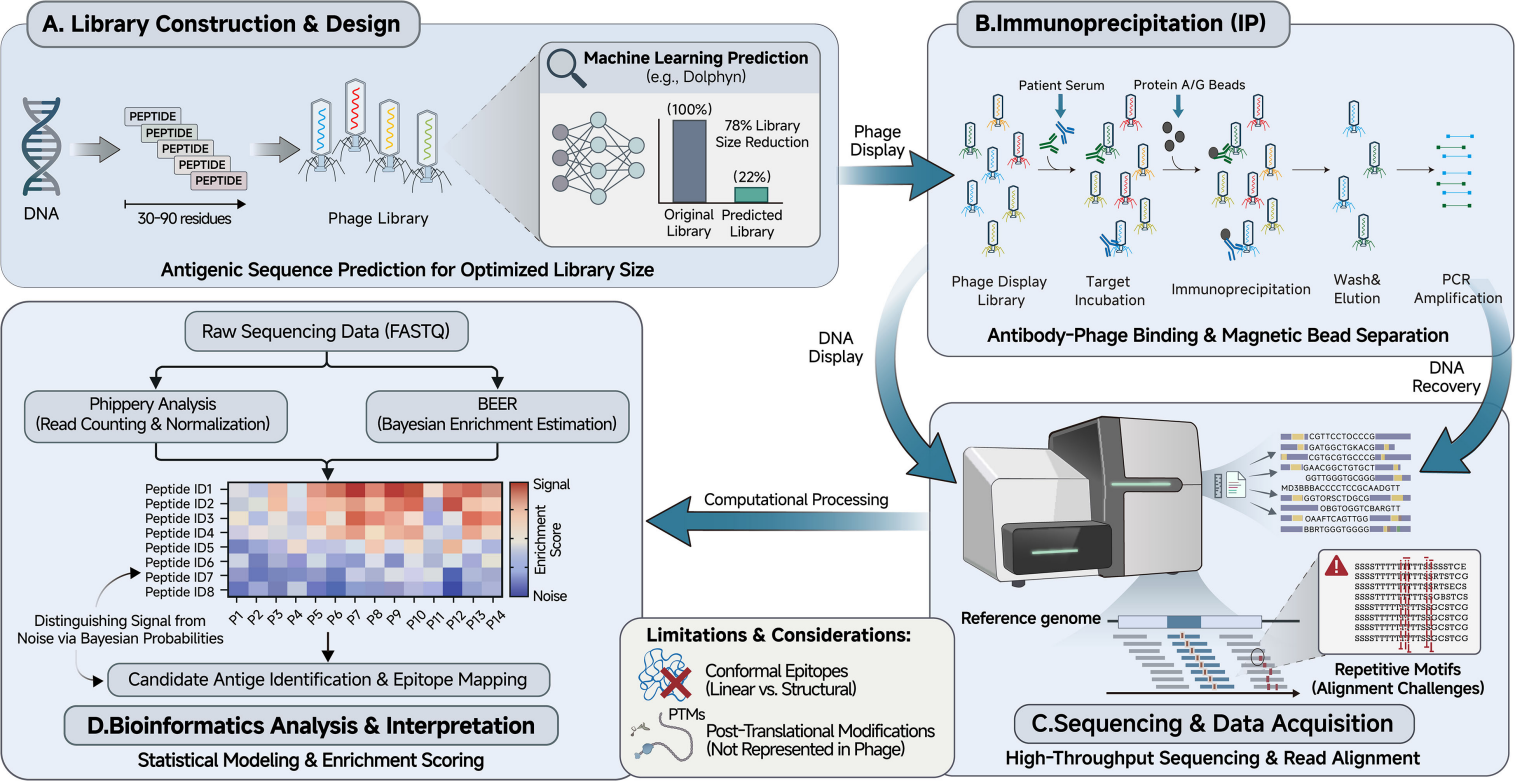
Overview of the PhIP-Seq workflow. The schematic illustrates the major steps of Phage-Immunoprecipitation Sequencing (PhIP-Seq), including **(A)** design and construction of oligonucleotide-encoded peptide libraries displayed on T7 bacteriophage, with optimization strategies such as machine learning-based epitope prediction; **(B)** immunoprecipitation of antibody-phage complexes using Protein A/G magnetic beads; **(C)** next-generation sequencing of enriched phage DNA; and **(D)** bioinformatics analysis pipeline incorporating alignment, normalization, and statistical enrichment modeling (e.g., BEER Bayesian framework). Key technical challenges and recent advancements are annotated throughout.

To provide a nuanced understanding of the platform, we examine the workflow steps alongside their associated technical hurdles and recent advancements, as shown in **Figure 1**.

### 2.1 Library design and construction

**Figure 2:**
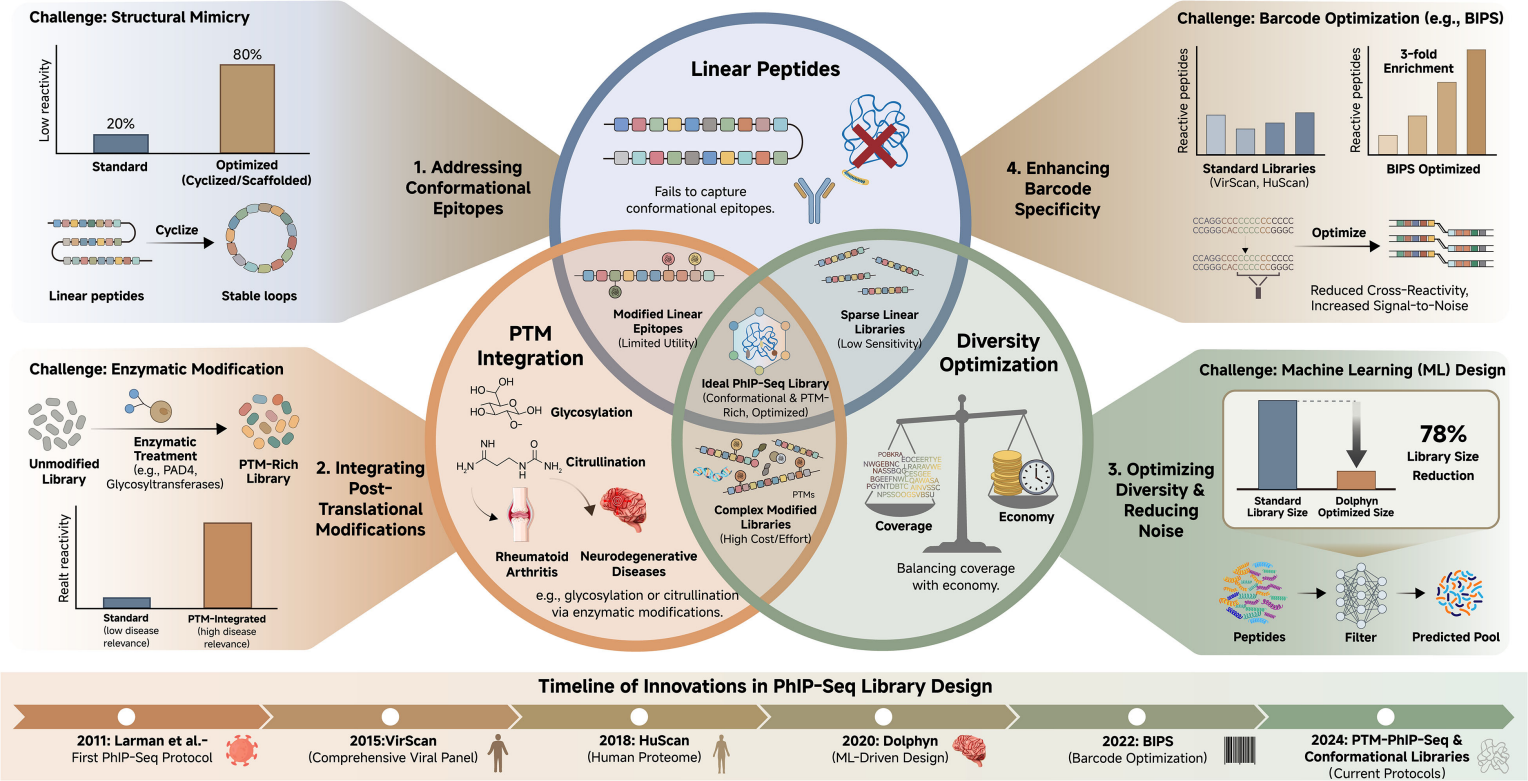
Challenges and optimization strategies in PhIP-Seq library design. A central Venn diagram depicts the primary limitations of standard linear peptide libraries: incomplete representation of conformational epitopes, lack of native post-translational modifications (PTMs), and trade-offs between comprehensiveness and library size. Radial extensions illustrate recent solutions, including machine learning tools for epitope prioritization (e.g., Dolphyn, achieving ∼78% library reduction with enhanced reactivity), barcode optimization, and incorporation of modified peptides (e.g., citrullination for rheumatoid arthritis-relevant antigens). Quantitative comparisons of library performance and timelines of key innovations are provided.

The fundamental component of PhIP-Seq is the oligonucleotide library, which encodes the antigens of interest. Standard libraries consist of peptides ranging from 30–90 residues, designed with overlapping sequences to ensure comprehensive epitope coverage (1, 12, 13).The design and optimization of these libraries involve navigating key trade-offs and addressing inherent challenges, a conceptual overview of which is provided in **Figure 2**. In practice, reference protein sequences are computationally tiled into fixed-length peptide “blocks” with a defined overlap (often ~half the tile length), then converted into DNA inserts that are codon-optimized and framed for display as fusions in a T7-based system (1, 3).

Several design choices strongly affect downstream interpretability. First, adapter/index strategy is usually implemented after immunoprecipitation: enriched library inserts are PCR-amplified with sequencing adapters and multiplexing indices to enable pooled NGS while preserving maximal insert capacity during oligo synthesis and cloning (1). Second, when restriction/ligation cloning is used, oligos are flanked with appropriate cloning arms and the encoded sequences are screened/optimized to avoid internal restriction sites, premature stops, and other problematic motifs that reduce cloning fidelity; T7 peptidome-style libraries and related programmable phage display systems commonly employ restriction-based insertion schemes (e.g., EcoRI/XhoI- or EcoRI/SalI-compatible designs) (14, 15). Third, uniqueness and unambiguous mapping are enforced by removing redundant tiles/near-duplicates at the peptide level and, where variants must be represented, by encoding synonymous DNA differences or internal barcodes that support accurate read identification without requiring full-length sequencing (16). Finally, rigorous quality control is essential to verify that the constructed library matches the intended design. Deep sequencing of the naïve library is routinely used to quantify representation (fraction of designed members observed), uniformity (abundance distribution), and complexity, and to detect bottlenecks introduced during amplification or packaging.
Synthesis and Vector Integration: Oligonucleotide library synthesis (OLS) allows for the creation of “mega-diverse” sets, such as VirScan (viral diversity) and HuScan (human proteome) (17). These sequences are cloned into phage vectors, with the T7 bacteriophage system being preferred over M13 due to its superior capacity for displaying high-complexity libraries with reduced bias (18–20).
Optimization Strategies: A critical challenge in library design is balancing coverage with economy (21). Recent innovations utilize machine learning algorithms to predict antigenic sequences, thereby reducing library size without sacrificing sensitivity (22, 23). For example, the tool Dolphyn uses epitope prediction to shrink libraries by 78% while enriching reactive peptides threefold (7, 24). Similarly, the BIPS software suite integrates barcode optimization to ensure error-resistant identification during downstream analysis (25, 26).To further enhance reproducibility, some protocols incorporate unique molecular identifiers (UMIs) during library construction, which helps distinguish true signals from PCR duplicates and sequencing errors.
Limitations (Conformational Epitopes & PTMs): A primary limitation of the standard library design is the reliance on linear peptides, which may fail to capture conformational epitopes dependent on 3D protein structure (5, 27, 28). Furthermore, the standard framework does not inherently incorporate post-translational modifications (PTMs) such as glycosylation or citrullination (26). To address this, recent protocols have introduced enzymatic or synthetic modifications to phage libraries to detect antibodies against modified motifs, such as citrullinated peptides in rheumatoid arthritis (7), though these methods increase experimental complexity.

### 2.2 Immunoprecipitation and phage display

Following library construction, the phage display and immunoprecipitation (IP) steps forge the link between genotype and phenotype.

The IP Workflow: The displayed library is incubated with patient serum or plasma, allowing antibodies to bind their cognate peptides (11, 29). After capture, wash stringency and buffer composition are tuned to retain specific antibody–peptide interactions while minimizing carryover of unbound phage, and the enriched phage pool is then recovered for downstream DNA amplification and sequencing-based readout (3).Antibody-phage complexes are subsequently captured using magnetic beads coated with Protein A or Protein G (30). Because the binding properties of Protein A and Protein G are strongly isotype dependent, this approach primarily enriches IgG antibodies under standard conditions. Protein A binds human IgG1, IgG2, and IgG4 with high affinity but exhibits weak binding to IgG3, whereas Protein G binds all human IgG subclasses while showing little to no affinity for IgM or IgA (8, 31, 32). Consequently, PhIP-Seq experiments relying on Protein A/G immunoprecipitation predominantly interrogate IgG-mediated antibody repertoires. The PhIP-Seq workflow can be modified for isotype-specific analyses by replacing Protein A/G beads with streptavidin-coated magnetic beads loaded with biotinylated isotype-specific capture antibodies. This approach has been applied in VirScan studies to enable IgA-specific immunoprecipitation, demonstrating that antibody profiling beyond IgG is technically achievable within the PhIP-Seq framework (33). For IgM, although dedicated PhIP-Seq applications remain limited in the published literature, isotype-specific capture using biotinylated anti-IgM antibodies is a well-established immunochemical strategy, supporting the conceptual extension of PhIP-Seq to IgM-focused analyses.

The same isotype-specific logic extends to IgE. In AllerScan, Monaco et al. profiled allergen-reactive antibodies with a T7 phage display library and captured IgE-bound phage using streptavidin magnetic beads loaded with biotinylated omalizumab, rather than Protein A/G. This design reflects the low abundance of serum IgE and the limited utility of standard Protein A/G pull-down conditions for IgE-focused readouts (34).In a later AllerScan application in peanut oral immunotherapy, IgE and IgG epitopes were measured side by side, illustrating that parallel isotype-resolved profiling can be implemented within the same phage-display sequencing workflow when clinically relevant (35).

Addressing Non-Specific Binding: Non-specific interactions between the phage/beads and serum components can generate significant background noise (36–39). Robust experimental design requires the inclusion of “mock” immunoprecipitations (no serum) and pre-immune samples to establish baseline noise levels (4, 40, 41). In practice, beads-only/mock IPs (no serum/antibody input) are typically processed in parallel to empirically define plate-specific background capture, and these negative controls can be incorporated into downstream statistical frameworks that explicitly model background distributions and improve discrimination of true enrichment from noise (42, 43).

### 2.3 Sequencing and bioinformatics

The final phase involves PCR amplification of the enriched phage DNA using sample-specific barcodes, followed by Next-Generation Sequencing (NGS) (44, 45).

Sequencing Platforms: While Illumina platforms remain the standard for high-throughput short-read sequencing, long-read technologies from PacBio and Oxford Nanopore are emerging as alternatives (46–48). These platforms can accommodate longer peptide coding sequences and reduce alignment errors associated with repetitive motifs (49–51).
Data Analysis and Standardization: The high volume of data-often millions of reads per sample-necessitates rigorous computational processing. Raw reads are aligned to the reference library and normalized against controls. Tools such as Phippery offer automated pipelines for alignment and counts matrix generation (52). To distinguish true enrichment from noise, Bayesian frameworks like BEER (Bayesian Estimation of Enrichment in PhIP-Seq) have been developed to model background distributions and assign posterior probabilities to antibody-peptide interactions (43). Standardization of these bioinformatic workflows remains a priority to ensure reproducibility across different cohorts (53).

In addition to technical standardization, several large-scale PhIP-Seq/VirScan studies indicate that inter-individual genetic variation can shape apparent enrichment patterns at the epitope level, particularly through the HLA region. Population cohorts integrating genotypes with PhIP-Seq readouts have reported reproducible associations between peptide reactivity and host loci including HLA, consistent with a measurable heritable component of epitope selection (54). Similar genetic effects have also been observed in specialized antigen libraries (e.g., toxin/virulence-factor panels), where MHC class II variation modulates bacterial epitope selection (55). Recent work further suggests that the detectability of HLA-II–linked antibody specificities depends on antigen properties, with secreted proteins and small antigen modules (e.g., viruses) showing more frequent HLA-II associations than large, complex antigen sets (56). Accordingly, when comparing cohorts or inferring exposure- or disease-associated signatures, incorporating ancestry/population structure covariates and, where available, HLA genotype as stratification variables can improve reproducibility and reduce misattribution of genetically driven reactivity differences to clinical effects (55).

## 3 Clinical applications

**Figure 3:**
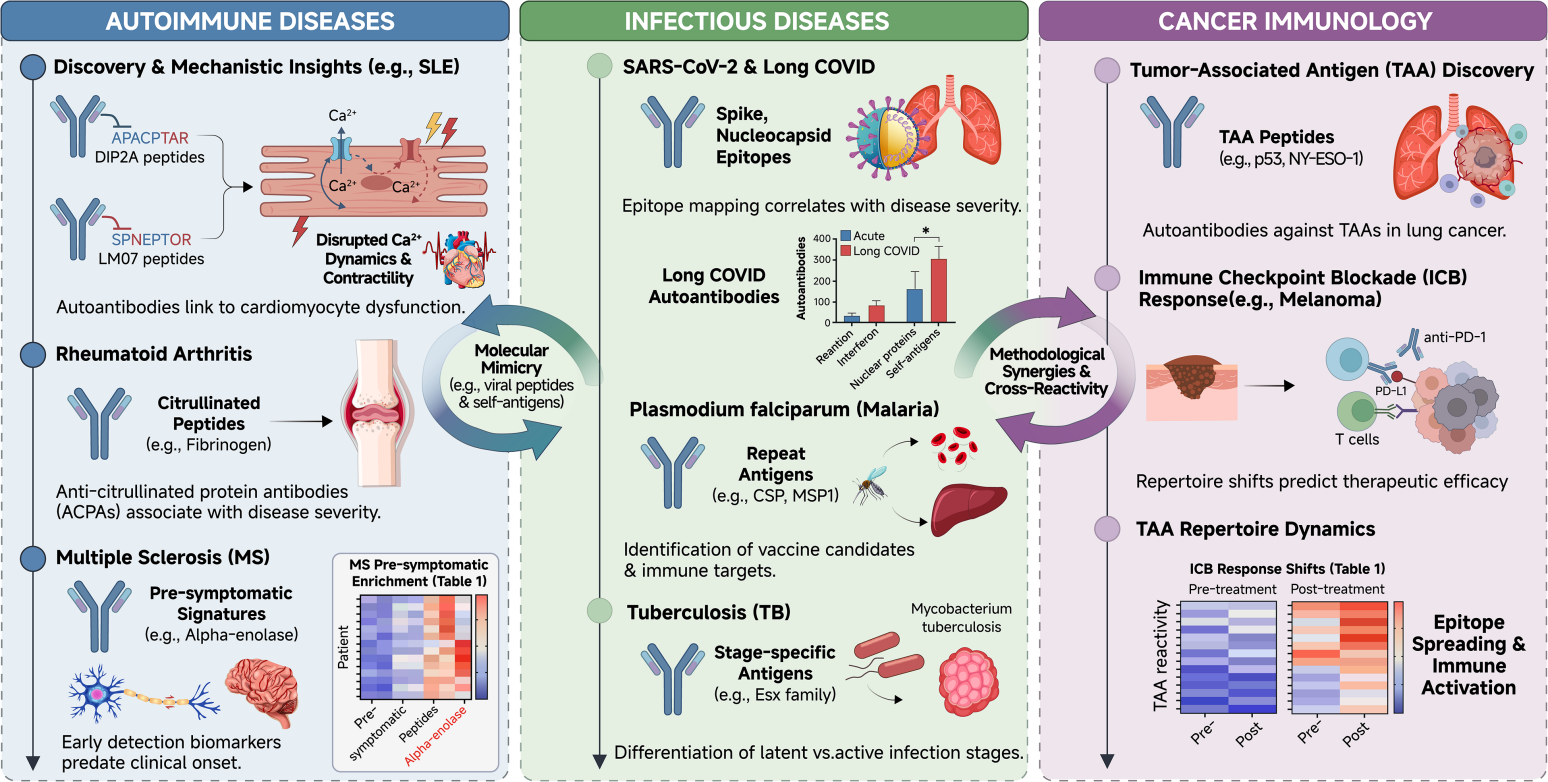
Clinical applications of PhIP-Seq across immunological domains. The landscape diagram categorizes major applications into autoimmune diseases, infectious diseases, and cancer immunology. Representative findings are highlighted for each domain, including identification of functionally relevant autoantibodies in systemic lupus erythematosus (e.g., anti-DIP2A/LMO7 linked to cardiomyocyte dysfunction), epitope mapping in SARS-CoV-2 and Plasmodium falciparum, pre-symptomatic signatures in multiple sclerosis, and tumor-associated antigens (e.g., p53, NY-ESO-1) as potential biomarkers. Overlaps indicate shared methodological insights and cross-reactive antibody responses.

The adaptability of PhIP-Seq has enabled its integration into three primary domains of immunological research, as shown in **Table 1** and **Figure 3**.

**Table 1:** Representative applications of PhIP-Seq and clinical implications.

Application domain	Disease/pathogen	Key findings & clinical relevance	Reference
Autoimmune Diseases	Systemic Lupus Erythematosus (SLE)	Identified autoantibodies (anti-DIP2A, anti-LMO7) that directly impair cardiomyocyte calcium handling, linking autoimmunity to heart failure.	(57)
	Rheumatoid Arthritis (RA)	Characterized fine specificity of antibodies to citrullinated peptides (ACPA), enhancing molecular diagnostics.	(58)
	Multiple Sclerosis (MS)	Defined a pre-symptomatic autoantibody signature (e.g., anti-alpha-enolase) enabling early prediction of disease onset.	(59)
Infectious Diseases	SARS-CoV-2	Mapped epitopes correlating with severe outcomes and identified autoantibodies persisting in Long COVID patients.	(62)
	Malaria (*P. falciparum*)	Profiled exposure-dependent antibodies to repeat-containing antigens to guide vaccine candidate selection.	(63)
Cancer Immunology	Lung Cancer/Scleroderma	Detected antibodies against tumor antigens p53 and NY-ESO-1; identified distinct scleroderma subtypes with coincident cancer risk.	(65)

### 3.1 Autoimmune diseases

In autoimmunity, PhIP-Seq excels at defining the precise targets of self-reactive antibodies.

Systemic Lupus Erythematosus (SLE): Recent studies have utilized PhIP-Seq to identify autoantibodies targeting proteins such as DIP2A, LMO7, and PVR. Crucially, research by Fleischer et al. (2024) demonstrated that these antibodies can disrupt cardiomyocyte calcium dynamics and mitochondrial integrity, providing a mechanistic link between serology and clinical heart failure (57), this mechanistic insight was derived from a focused cohort of 14 participants (11 patients stratified by cardiac involvement and 3 controls), demonstrating how deep phenotyping in a modest sample can elucidate pathogenic mechanisms. The study employed a standard, unmodified human proteome library, reflecting a classic discovery-oriented approach.
Rheumatoid Arthritis (RA): In RA, modified phage libraries have successfully profiled responses to citrullinated peptides, identifying them as foci for Anti-Citrullinated Protein Antibodies (ACPA), Roman-Meléndez et al. utilized a PTM-PhIP-Seq strategy to profile anti-citrullinated protein antibodies (ACPAs) (58). Their cohort included 56 participants (RA patients and healthy controls). Crucially, they employed a triad of libraries: a standard human proteome library, plus parallel libraries enzymatically modified by PAD2 or PAD4. This design enabled the direct identification and fine mapping of citrullination-dependent antibodies, revealing preferences for specific enzyme-modified epitopes. This represents a fundamental divergence from studies using only unmodified libraries, which would entirely miss this major class of RA autoantibodies.
Multiple Sclerosis (MS):In Multiple Sclerosis (MS), Zamecnik et al. identified a predictive autoantibody signature targeting antigens like alpha-enolase that appears pre-symptomatically, suggesting potential for early diagnostic screening (59),this finding is notable for its cross-cohort consistency. It was first discovered in a large, longitudinal Department of Defense Serum Repository (DoDSR) cohort of 500 individuals. The signature was then validated in an independent, cross-sectional ORIGINS cohort of 103 early MS patients and controls, with the signal detected in both serum and cerebrospinal fluid. Both cohorts were screened with the same human proteome-wide T7 phage library. The replication across distinct cohort types (pre-symptomatic military and clinical inception) and sample matrices underscores the robustness of this biomarker signal.
Rare Syndromes: The platform has also characterized autoantigens in monogenic syndromes like APS1 and paraneoplastic syndromes. Mandel-Brehm et al. identified ZSCAN1 as a target autoantigen in pediatric paraneoplastic ROHHAD (60),this study, involving 9 patients and multiple control groups, unveiled a critical factor affecting cross-methodological consistency. While PhIP-Seq and orthogonal assays (RLBA, CBA) confirmed ZSCAN1 reactivity, traditional immunohistochemistry on rodent brain tissue failed completely. The explanation lies in species specificity—the ZSCAN1 gene is absent in rodents. This case powerfully illustrates how the human-centric design of PhIP-Seq libraries can reveal autoantigens that are invisible to traditional, non-human antigen-based assays.

### 3.2 Infectious diseases

PhIP-Seq allows for multiplexed profiling of pathogen responses. The VirScan library, containing over 93,000 peptides from >200 viruses, has been pivotal in distinguishing between past and present infections (61).

SARS-CoV-2: During the COVID-19 pandemic, PhIP-Seq was used to map epitopes that correlate with disease severity and to identify autoantibodies associated with “Long COVID” sequelae (62).

Global Health Targets: In malaria research, the platform has identified *Plasmodium falciparum* epitopes relevant for vaccine design (63). Similarly, it has been applied to differentiate active from latent tuberculosis infection (64).

### 3.3 Cancer immunology

In oncology, PhIP-Seq serves as a tool for discovering Tumor-Associated Antigens (TAAs).

Biomarker Discovery: The method has detected autoantibodies against p53 and NY-ESO-1 in lung cancer patients, serving as potential early diagnostic markers (65).
Immune Checkpoint Blockade: In melanoma patients treated with checkpoint inhibitors, PhIP-Seq has been used to track shifts in antibody repertoires, correlating specific signatures with therapeutic outcomes and immune-related adverse events (66).

## 4 Future directions

**Figure 4:**
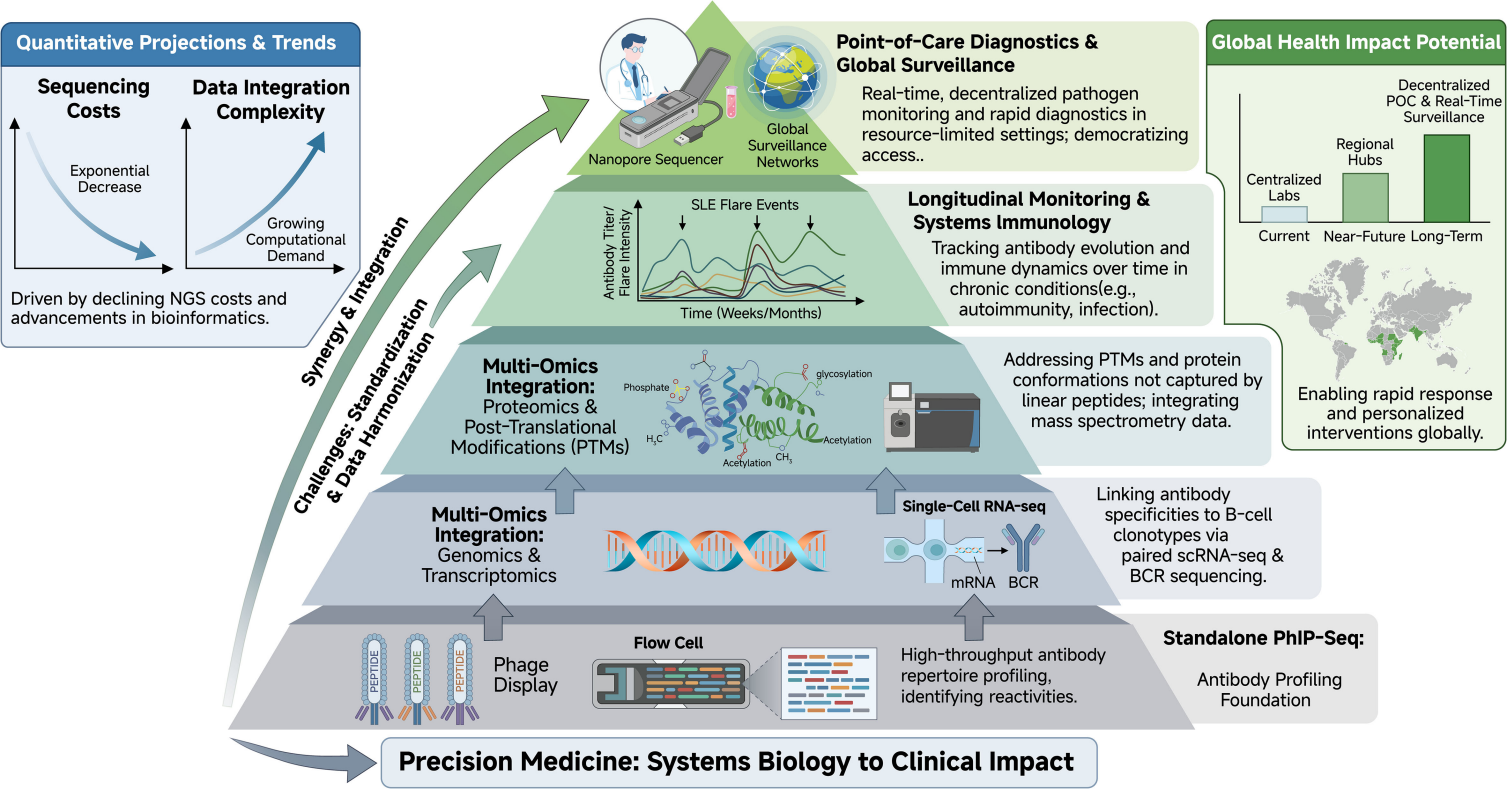
Future directions and integration of PhIP-Seq into multi-omics and precision medicine frameworks. A hierarchical pyramid structure depicts the progressive evolution of PhIP-Seq: from standalone high-throughput antibody profiling (base layer) to integration with genomics, transcriptomics, and proteomics for systems-level understanding; longitudinal monitoring for prognostic biomarker discovery; and translation to point-of-care diagnostics using portable sequencing platforms. Key synergies, such as linking antibody specificities to B-cell clonotypes via single-cell sequencing and addressing PTM-dependent responses, are emphasized, alongside anticipated impacts on personalized medicine and global health surveillance.

To fully realize the potential of PhIP-Seq, the field is moving toward integration with broader data ecosystems and clinical translation, as shown in **Figure 4**.

### 4.1 Multi-omics integration

Isolating antibody profiles provides only one dimension of the immune response. The integration of PhIP-Seq data with genomics, transcriptomics, and proteomics is essential for a systems-biology approach (67). For example, pairing PhIP-Seq with single-cell RNA sequencing can link antibody specificities to distinct B-cell clonotypes, clarifying the cellular origins of the humoral response (68). Furthermore, integrating proteomics can help elucidate PTM-dependent binding patterns that standard PhIP-Seq might miss (69).

### 4.2 Longitudinal monitoring and precision medicine

Longitudinal studies utilizing PhIP-Seq are crucial for understanding the temporal evolution of immunity. As demonstrated in COVID-19 cohorts, tracking antibody repertoires over time can reveal the persistence of immune perturbations (70). Scaling these longitudinal analyses to chronic autoimmune conditions will be vital for developing prognostic biomarkers that predict disease flares or therapeutic responses, moving the field toward personalized medicine (71).

### 4.3 Point-of-care diagnostics and surveillance

Advances in sequencing technologies are reducing the barrier to entry for PhIP-Seq. The adoption of portable sequencing platforms, such as Oxford Nanopore, offers the potential to miniaturize the workflow (72). This evolution could transition PhIP-Seq from centralized reference laboratories to point-of-care settings, facilitating rapid pathogen surveillance and real-time monitoring of autoimmune activity (73). Such capabilities would be particularly valuable for global health surveillance networks, allowing for the concurrent monitoring of multiple pathogens in resource-limited settings (74).

## 5 Conclusion

PhIP-Seq represents a fundamental shift in serological analysis. It has enabled researchers to systematically reveal previously inaccessible antibody interactions, including cross-reactive and low-affinity bindings that traditional assays lack the scale to detect. By merging scalable phage display with next-generation sequencing, the platform has greatly expanded the availability and broadened the adoption of high-dimensional immunological data in both research and clinical contexts.

Beyond its utility as a biomarker discovery tool, PhIP-Seq is increasingly driving mechanistic insights into disease pathology. The recent characterization of autoantibodies in systemic lupus erythematosus, which were shown to directly impair cardiomyocyte function, serves as a prime example of how the platform is moving from descriptive serology to functional immunology. This evolution is being accelerated by the maturation of computational frameworks like BEER and Phippery. These tools do more than just process data; they provide the statistical rigor necessary to deconvolve biological signals from the inherent noise of phage libraries, ensuring that high-throughput outputs translate into reproducible clinical intelligence.

Looking ahead, the true potential of PhIP-Seq lies in its integration into multi-omic systems biology. Correlating antibody specificities with host genetics and single-cell transcriptomics will be essential for deconstructing the heterogeneity seen in autoimmune and oncological cohorts. While technical hurdles—most notably the detection of complex conformational epitopes and the standardized incorporation of post-translational modifications—remain significant, the trajectory of the field is clear. As library designs become more sophisticated and sequencing costs continue to decline, PhIP-Seq is poised to become an indispensable component of precision medicine, offering a high-resolution lens through which we can finally understand the full complexity of the human immune response in health and disease.
